# Association Analysis of Dyslipidemia-Related Genes in Diabetic Nephropathy

**DOI:** 10.1371/journal.pone.0058472

**Published:** 2013-03-26

**Authors:** Gareth J. McKay, David A. Savage, Christopher C. Patterson, Gareth Lewis, Amy Jayne McKnight, Alexander P. Maxwell, the Warren 3/UK GoKinD Study Group

**Affiliations:** 1 Nephrology Research Group, Centre for Public Health, Queen's University Belfast, Belfast, United Kingdom; 2 Histocompatibility & Immunogenetics Laboratory, Belfast Health & Social Care Trust, Belfast City Hospital, Belfast, United Kingdom; 3 Cardiovascular Epidemiology and Public Health Research Group, Centre for Public Health, Queen's University Belfast, Belfast, United Kingdom; University of Siena, Italy

## Abstract

Type 1 diabetes (T1D) increases risk of the development of microvascular complications and cardiovascular disease (CVD). Dyslipidemia is a common risk factor in the pathogenesis of both CVD and diabetic nephropathy (DN), with CVD identified as the primary cause of death in patients with DN. In light of this commonality, we assessed single nucleotide polymorphisms (SNPs) in thirty-seven key genetic loci previously associated with dyslipidemia in a T1D cohort using a case-control design. SNPs (n = 53) were genotyped using Sequenom in 1467 individuals with T1D (718 cases with proteinuric nephropathy and 749 controls without nephropathy i.e. normal albumin excretion). Cases and controls were white and recruited from the UK and Ireland. Association analyses were performed using PLINK to compare allele frequencies in cases and controls. In a sensitivity analysis, samples from control individuals with reduced renal function (estimated glomerular filtration rate<60 ml/min/1.73 m^2^) were excluded. Correction for multiple testing was performed by permutation testing. A total of 1394 samples passed quality control filters. Following regression analysis adjusted by collection center, gender, duration of diabetes, and average HbA1c, two SNPs were significantly associated with DN. rs4420638 in the *APOC1* region (odds ratio [OR]  = 1.51; confidence intervals [CI]: 1.19–1.91; P = 0.001) and rs1532624 in *CETP* (OR = 0.82; CI: 0.69–0.99; P = 0.034); rs4420638 was also significantly associated in a sensitivity analysis (P = 0.016) together with rs7679 (P = 0.027). However, no association was significant following correction for multiple testing. Subgroup analysis of end-stage renal disease status failed to reveal any association. Our results suggest common variants associated with dyslipidemia are not strongly associated with DN in T1D among white individuals. Our findings, cannot entirely exclude these key genes which are central to the process of dyslipidemia, from involvement in DN pathogenesis as our study had limited power to detect variants of small effect size. Analysis in larger independent cohorts is required.

## Introduction

Type 1 diabetes mellitus (T1D) has been previously reported to increase the risk of microvascular complications and cardiovascular disease (CVD) [1]–[3]. In contrast to the reduction in cardiovascular mortality within the general US population, the declining trend is less evident in individuals with diabetes [4]. Despite improved disease management strategies, CVD remains the primary cause of death in patients with T1D [5] and a ten-fold increase in risk is reported in those with diabetic nephropathy (DN) relative to those without it [6]. DN is a complex, multi-factorial disease and identifying robust genetic risk factors has proved challenging. Several risk factors are common to both CVD and DN, including hypertension, male gender, smoking and modifiable dyslipidemia [5]–[11].

Dyslipidemia results from abnormal lipid metabolism with departure from optimum vascular cholesterol and triglyceride levels leading to atherosclerosis, a process of fatty acid plaque deposition in arterial blood vessels. Previous studies reported normal low-density lipoprotein (LDL) and high-density lipoprotein (HDL) cholesterol levels in individuals with T1D, with elevated triglyceride levels more commonly associated with poor glycemic control [12]. This abnormal lipid profile can result from insulin deficiency with subsequent reduction in lipoprotein lipase activity and diminished ability for chylomicron and very low-density lipoprotein (VLDL) clearance [13]. This contrasts with individuals with type 2 diabetes (T2D) who often exhibit reduced HDL levels with a shift in LDL to the more atherogenic dense VLDL particles as a consequence of increased hepatic production. This process is increased by insulin resistance resulting in reduced clearance of VLDL and chylomicrons [14].

Observational studies have identified multiple lipid abnormalities in both incipient and overt DN [15]–[17], although this has not been consistently reported [18]. While the exact mechanism of effect is not fully understood, dyslipidemia has been associated with DN progression as well as increasing cardiovascular risk [19]–[20]. Supporting evidence implicates insulin resistance as pivotal in the development and/or progression of this condition [21]–[25]. Potential mechanisms contributing to renal injury in DN have included stimulation of pro-inflammatory and pro-fibrotic cytokine production, cell apoptosis, vasoconstriction and modulation of mesangial cell proliferation [26]–[28]. As such, parallels between mechanisms that underpin atherosclerosis and glomerulosclerosis provide support for investigation of the parameters that contribute to both conditions [29].

While previous evidence demonstrates modulation of lipid profiles through lifestyle changes such as smoking, diet and physical activity, recent studies have also identified common genetic variation as regulators of lipid levels and subsequent dyslipidemia [30]–[37]. To date, almost 100 genetic loci have been reported in association with serum cholesterol and triglyceride levels [38]. Aulchenko and colleagues highlighted that many of the loci influencing lipid levels and CVD risk had previously been identified in association studies enriched by participants with diabetes [34]. The management of diabetic dyslipidemia, a well-recognized and modifiable risk factor, is a key element in the multifactorial approach to prevent CVD in individuals with diabetes [23]. In light of the evidence supporting association of these variants with dyslipidemia in individuals with diabetes, we sought to assess the allelic frequency of 53 common single nucleotide polymorphisms (SNPs) in 37 key loci in individuals with DN using a case-control design involving 1467 individuals with T1D. These loci and SNPs were selected on the basis of their functional significance and previous reported association with dyslipidemia [30]–[37].

## Methods

### Participants

Research ethics approval was obtained from the South and West Multicentre Research Ethics Committee (MREC/98/6/71) and Queens University Belfast Research Ethics Committee. Written informed consent was obtained prior to participation. All recruited individuals were white, had T1D diagnosed before 32 years of age and were born in the UK or Ireland. Patients (n = 718) and controls (n = 749) originated from the Warren 3/UK Genetics of Kidneys in Diabetes (GoKinD) and all-Ireland collections [39]. The definition of DN in cases was based on development of persistent proteinuria (>0.5 g protein/24 h) at least 10 years after diagnosis of T1D, hypertension (blood pressure >135/85 mmHg or treatment with antihypertensive agents) and associated diabetic retinopathy. Controls were individuals with T1D for at least 15 years with normal urinary albumin excretion rates and no evidence of microalbuminuria on repeated testing. In addition, control subjects had not been prescribed antihypertensive drug treatment avoiding possible misclassification of diabetic individuals as ‘control phenotypes’ when the use of antihypertensive treatment may have reduced urinary albumin excretion into the normal range. Individuals with microalbuminuria were excluded from both case and control groups since it was not possible to be confident in assigning case/control status for such individuals whose urinary albumin excretion might either regress or progress over time [40].

### SNP selection and genotyping

SNPs (n = 53) were selected on the basis of previously reported association with dyslipidemia [31]–[34] and of minor allele frequency (MAF) exceeding 0.1 in populations of European descent. Genotyping was performed by MassARRAY iPLEX (Sequenom, San Diego, CA, USA) assays according to the manufacturer's instructions. Quality filters for exclusion of SNPs included call rates below 95% and deviation from HWE (*P*<0.001). DNA samples were excluded if missing genotypes exceeded 10%. Other quality control measures included parent/offspring trio samples, duplicates on plates, random sample allocation to plates, independent scoring of problematic genotypes by two individuals and re-sequencing of selected DNAs to validate genotypes.

### Statistical analysis

Clinical characteristics of cases and controls were compared using the z-test for large independent samples and the χ^2^ test. Association analyses were performed using PLINK (version 1.07; http://pngu.mgh.harvard.edu/~purcell/plink/). Initially a χ^2^ test for trend (1 *df*) was used with stratification by collection center. Logistic regression analysis was performed on each SNP with terms for potential confounders (collection center, gender, duration of T1D and HbA1c) included in the model. A sensitivity analysis to minimize potential misclassification of case/control status was performed by excluding samples from those control individuals with an estimated glomerular filtration (eGFR) <60 ml/min/1.73 m^2^. The level of statistical significance was set at 5% and adjustment for multiple testing performed by permutation test (n = 100,000). Potential gene-gene interactions between SNPs were assessed using likelihood ratio χ^2^ tests in the logistic regression with terms for potential confounders (collection center, gender, duration of T1D and HbA1c) included in the model.

## Results

The clinical characteristics of the DN cases (n = 718) and diabetic controls (n = 749) genotyped in this study are listed in Table 1. There were more males, higher mean HbA1c and blood pressure values (despite the use of antihypertensive treatment) in the case group compared with the control group. All comparisons were significant at *P*<0.001 with the exception of age at diagnosis, LDL cholesterol and body mass index which did not differ significantly between groups. Approximately one quarter of cases (26.9%) had end-stage renal disease (ESRD).

**Table 1:** Clinical characteristics of diabetic nephropathy (DN) cases and no nephropathy diabetic controls.

Characteristic	DN cases (n = 718)	Controls (n = 749)	P value
Male; n (%)	415 (57.8%)	320 (42.7%)	<0.001
Age at diagnosis of T1D (yr)	14.8±7.7	15.5±7.9	0.09
Duration of T1D (yr)^a^	33.3±9.4	28.1±9.0	<0.001
Age at sampling	48.1±10.4	43.6±11.0	<0.001
HbA1c (%)^b^	9.0±1.9	8.6±1.5	<0.001
Systolic blood pressure (mmHg)^b^	144.9±20.9	125.0±14.7	<0.001
Diastolic blood pressure (mmHg)^b^	81.5±11.4	75.4±7.8	<0.001
Body mass index (kg/m^2^)	26.3±4.7	26.1±4.2	0.50
Serum cholesterol (mmol/L)	5.34±1.22	5.09±0.91	<0.001
HDL cholesterol (mmol/L)	1.59±0.55	1.78±0.47	<0.001
LDL cholesterol (mmol/L)	2.88±0.95	2.80±0.75	0.17
Serum triglycerides (mmol/L) median (interquartile range)	1.4 (1.0–2.2)	1.0 (0.7–1.4)	<0.001
Serum creatinine (µmol/L);^c^ median (interquartile range)	130 (102–183)	92 (79–105)	<0.001
Estimated glomerular filtration rate (ml/min/1.73m^2^);^c^ median (interquartile range)	48 (33–66)	70 (59–85)	<0.001
End-stage renal disease; n (%)	193 (26.9%)	NA	NA
Passed quality control criteria; n (%)	684 (95.3%)	710 (94.8%)	0.68

Unless otherwise stated values are mean ± standard deviation.

a Calculated from the dates of diagnosis and recruitment.

b Average of the three most recent values prior to recruitment.

c Excludes subjects receiving renal replacement therapy (dialysis or transplant).

A total of 53 SNPs were genotyped using MassARRAY iPLEX technology in 718 cases and 749 controls (Table 2). We excluded 73 samples (34 cases and 39 controls) from the analysis with ≥10% missing genotypes. The average call rate for all SNPs analysed was 99.3%. The genotype distribution for each SNP did not deviate significantly from HWE in either cases or controls. No duplicate or Mendelian inconsistencies were observed.

**Table 2:** Minor allele frequencies (MAF) and genotype counts in 684 diabetic nephropathy cases and 710 no nephropathy diabetic controls.

			Genomic				Case		Controls				Confidence		
SNP ID	Ref	C/some	Position	Variant	Gene	aAlleles	Counts	MAF	Counts	MAF	bP val	cOR	Interval	dP val	eP val
rs10903129	34	1	25768937	intronic	*TMEM57*	[A/G]	128/319/236	0.42	124/364/220	0.43	0.548	0.92	0.77–1.10	0.368	0.261
rs11206510	33	1	55496039	intergenic	*PCSK9*	[C/T]	32/172/461	0.18	25/217/464	0.19	0.431	0.90	0.72–1.12	0.341	0.377
rs1167998	34	1	62931632	intronic	*DOCK7*	[C/A]	84/298/298	0.34	83/308/314	0.34	0.719	0.98	0.82–1.18	0.867	0.957
rs10889353	34	1	63118196	intronic	*DOCK7*	[C/A]	81/297/305	0.34	80/307/320	0.33	0.748	0.98	0.82–1.18	0.858	0.957
1rs12740374	32	1	109817590	3′UTR	*CELSR2*	[T/G]	33/230/421	0.22	36/219/452	0.21	0.494	1.10	0.89–1.36	0.388	0.520
rs646776	32	1	109818530	intergenic	*CELSR2*	[G/A]	33/225/424	0.21	37/219/452	0.21	0.678	1.07	0.86–1.32	0.540	0.707
rs2144300	31	1	230294916	intronic	*GALNT2*	[C/T]	105/337/235	0.40	108/330/269	0.39	0.337	1.07	0.89–1.28	0.490	0.573
rs4846914	32	1	230295691	intronic	*GALNT2*	[G/A]	109/339/236	0.41	110/332/268	0.39	0.320	1.05	0.88–1.26	0.578	0.620
rs6754295	34	2	21206183	intergenic	*APOB*	[G/T]	27/238/416	0.21	39/239/432	0.22	0.573	1.02	0.83–1.27	0.833	0.318
rs7557067	33	2	21208211	intergenic	*APOB*	[G/A]	28/241/415	0.22	40/238/432	0.22	0.663	1.03	0.83–1.27	0.800	0.286
rs673548	34	2	21237544	intronic	*APOB*	[A/G]	20/228/430	0.20	30/215/454	0.20	0.951	1.07	0.85–1.34	0.564	0.263
rs1260326	33	2	27730940	missense	*GCKR*	[T/C]	93/329/258	0.38	114/305/290	0.38	0.879	0.99	0.83–1.19	0.943	0.642
rs780094	34	2	27741237	intronic	*GCKR*	[A/G]	86/311/273	0.36	104/296/294	0.36	0.885	0.98	0.82–1.18	0.842	0.798
rs6756629	34	2	44065090	missense	*ABCG5*	[A/G]	3/90/591	0.07	4/92/610	0.07	0.947	0.90	0.64–1.27	0.560	0.449
rs6544713	33	2	44073881	intronic	*ABCG8*	[T/C]	68/308/307	0.33	86/305/318	0.34	0.525	1.03	0.85–1.24	0.759	0.811
rs3846662	34	5	74651084	intronic	*HMGCR*	[C/T]	104/324/246	0.39	113/323/254	0.40	0.866	0.98	0.82–1.17	0.815	0.258
rs3846663	33	5	74655726	intronic	*HMGCR*	[T/C]	83/302/297	0.34	92/308/309	0.35	0.831	0.96	0.80–1.16	0.694	0.258
rs1501908	33	5	156398169	intergenic	*TIMD4/HAVCR1*	[C/G]	102/313/265	0.38	99/329/279	0.37	0.686	1.10	0.92–1.32	0.288	0.200
rs12670798	34	7	21607352	intronic	*DNAH11*	[C/T]	37/255/392	0.24	43/254/413	0.24	0.948	0.99	0.81–1.22	0.945	0.759
rs2240466	34	7	72856269	intronic	*BAZ1B*	[T/C]	5/175/503	0.14	14/151/545	0.13	0.463	1.13	0.87–1.47	0.374	0.109
2rs714052	32	7	72864869	intronic	*BAZ1B*	[C/T]	5/173/503	0.13	14/151/543	0.13	0.534	1.12	0.86–1.46	0.396	0.115
rs7819412	33	8	11045161	intronic	*XKR6*	[G/A]	168/322/179	0.49	152/361/196	0.47	0.231	1.05	0.88–1.26	0.583	0.420
rs10096633	34	8	19830921	intergenic	*LPL*	[T/C]	11/124/548	0.11	8/145/557	0.11	0.584	0.82	0.62–1.08	0.154	0.130
rs12678919	33	8	19844222	intergenic	*LPL*	[G/A]	9/97/576	0.08	5/111/593	0.09	0.923	0.84	0.62–1.14	0.265	0.219
rs2083637	34	8	19865175	intergenic	*LPL*	[C/T]	50/245/388	0.25	51/271/388	0.26	0.542	0.90	0.74–1.10	0.321	0.191
rs17321515	32	8	126486409	intergenic	*TRIB1*	[G/A]	153/343/188	0.47	153/365/191	0.47	0.949	1.03	0.86–1.23	0.717	0.564
3rs2954029	32	8	126490972	intergenic	*TRIB1*	[T/A]	149/333/202	0.46	147/366/197	0.46	0.852	1.02	0.86–1.22	0.797	0.617
rs471364	33	9	15289578	intronic	*TTC39B*	[G/A]	6/147/529	0.12	11/144/554	0.12	0.967	1.01	0.77–1.33	0.951	0.807
rs3905000	34	9	107657070	intronic	*ABCA1*	[A/G]	11/180/492	0.15	17/179/513	0.15	0.863	1.02	0.80–1.31	0.882	0.810
rs1883025	33	9	107664301	intronic	*ABCA1*	[A/G]	51/276/356	0.28	55/316/338	0.30	0.168	0.94	0.78–1.15	0.573	0.620
rs7395662	34	11	48518893	intergenic	*OR4A47*	[A/G]	95/326/263	0.38	92/339/279	0.37	0.628	1.08	0.90–1.30	0.415	0.524
rs174547	33	11	61570783	intronic	*FADS1*	[C/T]	78/319/287	0.35	70/327/306	0.33	0.402	1.13	0.93–1.37	0.212	0.524
rs174570	34	11	61597212	intronic	*FADS2*	[T/C]	8/161/508	0.13	10/168/525	0.13	0.817	1.04	0.79–1.35	0.799	0.527
rs964184	32	11	116648917	intergenic	*ZNF259*	[G/C]	10/138/535	0.12	10/162/538	0.13	0.314	0.82	0.63–1.07	0.142	0.172
rs2338104	31	12	109895168	intronic	*KCTD10*	[C/G]	149/340/193	0.47	189/329/189	0.50	0.089	0.91	0.76–1.08	0.268	0.172
rs2650000	33	12	121388962	intergenic	*HNF1A*	[T/G]	76/292/312	0.33	82/315/307	0.34	0.444	0.88	0.73–1.06	0.180	0.257
rs4775041	31	15	58674695	intergenic	*LIPC*	[C/G]	65/288/328	0.31	68/284/356	0.30	0.555	1.05	0.87–1.27	0.596	0.348
rs10468017	33	15	58678512	intergenic	*LIPC*	[T/C]	61/290/330	0.30	65/279/366	0.29	0.403	1.07	0.88–1.29	0.502	0.275
rs1532624	34	16	57005479	intronic	*CETP*	[T/G]	109/337/233	0.41	149/341/213	0.45	0.015	0.82	0.69–0.99	0.034	0.514
rs2271293	34	16	67902070	intronic	*NUTF2*	[A/G]	11/131/521	0.12	8/129/543	0.11	0.470	0.93	0.70–1.25	0.638	0.272
rs4939883	34	18	47167214	intergenic	*LIPG*	[T/C]	28/213/437	0.20	26/212/467	0.19	0.458	1.06	0.85–1.33	0.612	0.585
rs2967605	33	19	8469738	intergenic	*RAB11B*	[A/G]	25/207/446	0.19	24/214/468	0.19	0.789	1.08	0.86–1.35	0.526	0.717
rs6511720	33	19	11202306	intronic	*LDLR*	[T/G]	11/145/526	0.12	6/144/558	0.11	0.313	1.08	0.82–1.43	0.575	0.083
rs2228671	34	19	11210912	missense	*LDLR*	[T/C]	12/148/521	0.13	8/160/542	0.12	0.852	1.00	0.77–1.30	0.989	0.289
4rs10401969	32	19	19407718	intronic	*SUGP1*	[C/T]	5/91/587	0.07	4/101/605	0.08	0.778	0.93	0.67–1.29	0.667	0.415
5rs17216525	32	19	19662220	intergenic	*CILP2PBX4*	[T/C]	5/97/582	0.08	6/107/597	0.08	0.589	0.94	0.68–1.29	0.685	0.382
rs2304130	34	19	19789528	intronic	*ZNF101*	[G/A]	7/96/571	0.08	5/106/594	0.08	0.949	0.98	0.72–1.34	0.913	0.487
rs157580	34	19	45395266	intronic	*TOMM40*	[G/A]	122/319/242	0.41	121/345/241	0.42	0.873	1.01	0.84–1.21	0.916	0.626
rs2075650	34	19	45395619	intronic	*TOMM40*	[G/A]	11/163/508	0.14	10/184/514	0.14	0.522	1.01	0.78–1.30	0.956	0.877
rs439401	34	19	45414451	intergenic	*APOE/APOC1*	[T/C]	96/313/275	0.37	96/348/266	0.38	0.544	0.99	0.82–1.18	0.882	0.772
rs4420638	33	19	45422946	intergenic	*APOC1*	[G/A]	24/224/421	0.20	11/199/475	0.16	0.005	1.51	1.19–1.91	0.001	0.0160
rs6102059	33	20	39228784	intergenic	*MAFB*	[T/C]	68/312/304	0.33	65/327/318	0.32	0.750	0.96	0.80–1.17	0.713	0.499
rs7679	33	20	44576502	3′UTR	*PCIF1*	[C/T]	29/178/473	0.17	24/222/462	0.19	0.242	0.91	0.72–1.14	0.389	0.027

a Minor alleles are presented first followed by major allele.

b Unadjusted P values were calculated as tests for trend (1 df) across genotypes.

c Odds ratios and 95% confidence intervals are calculated on a per allele basis for the minor allele assuming an additive model.

d Adjusted P values were calculated as tests for trend (1 df) across genotypes in a logistic regression which included terms for collection center, gender, duration of T1DM and HbA1c category. Associations were no longer significant after correction for multiple testing performed by permutation test (n = 100,000).

e In a sensitivity analysis (Control samples only with eGFR>60 ml/min/1.73 m^2^; n = 444) adjusted P values were calculated as tests for trend (1 df) across genotypes in a logistic regression which included terms for collection center, gender, duration of T1DM and HbA1c category. Associations were no longer significant after correction for multiple testing performed by permutation test (n = 100,000).

1 Proxy for rs599839 (r^2^ = 0.90).

2 Proxy for rs17145738 (r^2^ = 1).

3 Proxy for rs17321515 (r^2^ = 0.97).

4 Proxy for rs16996148 (r^2^ = 0.90).

5 Proxy for rs16996148 (r^2^ = 1).

Single marker testing stratified by collection center identified two non-coding SNPs (rs1532624 in Cholesteryl ester transfer protein (*CETP*) and rs4420638 in Apolipoprotein C-I *APOC1*) to be significantly associated with DN (Table 2). In logistic regression analysis with adjustment by collection center, gender, duration of T1D, and average HbA1c as covariates, the significance of both SNPs was maintained (rs1532624: odds ratio [OR]  = 0.82; confidence intervals [CI]: 0.69–0.99; P = 0.034; rs4420638: OR = 1.51; CI: 1.19–1.91; P = 0.001). The sensitivity analysis (that includes samples only from those controls with eGFR >60 ml/min/1.73 m^2^) identified two SNPs significantly associated with DN in the fully adjusted model (rs4420638; P = 0.016 and rs7679; P = 0.027). However, no associations were maintained following correction for multiple testing. Subgroup analyses showed no association of any SNP with ESRD status.

With no prior hypotheses or supporting evidence of potential gene-gene interaction, we assumed a more stringent level of significance (P<0.01). Interactions were assessed using likelihood ratio χ^2^ tests in the logistic regression with terms for potential confounders (collection center, gender, duration of T1D and HbA1c) included in the model. Seven interaction terms exceeded the minimum threshold set but following correction for multiple testing and examination of the resultant Q-Q plot, none were identified as being worthy of further investigation (Table 3).

**Table 3:** Assessment of gene-gene pair-wise interactions.

SNP 1	Gene 1	SNP 2	Gene 2	^1^P value	^2^P value
rs3905000	*ABCA1*	rs7679	*PCIF1*	0.002	0.014
rs6756629	*ABCG5*	rs714052	*BAZ1B*	0.003	0.009
rs2240466	*BAZ1B*	rs6756629	*ABCG5*	0.007	0.01
rs2240466	*BAZ1B*	rs12678919	*LPL*	0.007	0.115
rs1167998	*DOCK7*	rs17216525	*CILP2PBX4*	0.008	0.002
rs10903129	*TMEM57*	rs6544713	*ABCG8*	0.009	0.024
rs12678919	*LPL*	rs714052	*BAZ1B*	0.009	0.14

The number of significant interactions observed is less than one might expect by chance.

P values for gene-gene interactions were obtained between SNPs using likelihood ratio χ^2^ tests in the logistic regression. Data are presented for those which attained significance at the P<0.01 level in an unadjusted model^1^. Significance levels are also presented where terms for potential confounders (collection center, gender, duration of T1D and HbA1c) are included in the adjusted model^2^.

## Discussion

Dyslipidemia can result through dietary and lifestyle influences or alternatively as a consequence of variation in genes pivotal to lipoprotein metabolism. In persons with diabetes, prolonged elevation of insulin levels often leads to dyslipidemia, a process central to the pathogenesis of atherosclerosis and increasing CVD risk. As previous studies have reported multiple lipid abnormalities in patients with T1D [15]–[20], we evaluated common polymorphic variation previously associated with dyslipidemia, in persons with T1D, both with and without DN. Univariate analysis identified two SNPs associated with DN (rs1532624 in *CETP* and rs4420638 in *APOC1*) both of which remained significant following adjustment for collection center, gender, duration of T1D, and average HbA1c. Interestingly, rs4420638 was also significantly associated with DN in the sensitivity analysis using only those samples from diabetic controls with eGFR >60 ml/min/1.73 m^2^. However, following correction for multiple testing, these associations were no longer significant. Although, published data were available from the US GoKinD genome-wide association study, limited coverage on the Affymetrix 500 K genotyping platform across the genomic locations of both *CETP* and *APOC1*, prevented *in silico* independent replication or meta-analysis of our top SNPs or any potential proxies (r^2^>0.8) [41].

In previously published studies the definition of the DN phenotype has proved challenging. We do not think it is surprising that cases in our study had persistent proteinuria (macroalbuminuria) despite the use of antihypertensive medication. The use of angiotensin converting enzyme inhibitors (ACEi) or angiotensin receptor blockers (ARBs), typically reduces but does not abolish protein excretion in persons with overt diabetic nephropathy [42]–[44] suggesting that persistent proteinuria is unlikely to be a consequence of suboptimal blood pressure control. The differences in mean blood pressures observed between case and control groups were consistent with findings in clinical practice.

In addition, it has been suggested that some individuals with a very prolonged duration of type 1 diabetes may develop chronic kidney disease (CKD) without albuminuria. Molitch and colleagues [45] identified 89 of the 1,439 individuals recruited to the DCCT/EDIC study that had developed CKD (defined by estimated GFR <60 ml/min/1.73 m^2^) after almost 20 years of follow up. Of the 89 individuals with CKD, 21 were classified as normoalbuminuric (albumin excretion rate [AER] <30); 14 as microalbuminuric (AER: 30–300); and 54 as macroalbuminuric (AER >300). Of note 43% of the normoalbuminuric individuals with CKD were taking ACEi during the study and 14% were taking ARBs at year 13/14 of the EDIC study [45]. The antihypertensive drugs, ACEi and ARBs, can both lower AER and reduce eGFR which may partly explain why the authors found a small number of individuals with normoalbuminuria and reduced eGFR. The normoalbuminuric patients with reduced eGFR were also 4 years older at time of recruitment than the macroalbuminuric patients (30+/−7 yr vs. 26+/− 7 yr [45]). Nevertheless, we did attempt to address this issue of diabetic patients having CKD without albuminuria. In a sensitivity analysis, we excluded all those diabetic patients we had originally recruited as normalbuminuric controls in whom the eGFR was <60 ml/min/1.73 m^2^. We excluded these controls with reduced renal function from our analysis to limit any risk of misclassification of nephropathy status but found this made little difference to the main analysis (Table 2).

CETP is a protein central to the process of dyslipidemia. It acts as a mediator for the transfer of cholesteryl esters from HDL to VLDL or LDL in exchange for triglycerides, reducing serum HDL concentrations [46]. Variation in CETP levels have been correlated with lipid metabolism and insulin resistance in Type 2 diabetes [47], and also in association with the development of obesity [48] and susceptibility to atherosclerosis and other CVD [49]. Recently, Igl and colleagues demonstrated that the genetic influence mediated by rs1532624 could be attenuated by lifestyle factors such as diet or physical activity, highlighting the potential for interaction at this locus [50]. Our study was unable to examine lifestyle influences, as dietary and physical activity measurements were not collected during recruitment. Nonetheless we sought to evaluate the potential for pair-wise gene-gene interaction between the candidate SNPs examined. Several pair-wise interactions which included the *CETP* and *APOB* loci were identified but did not remain significant following correction for multiple testing. As no association survived correction for multiple testing, it is unlikely these gene variants play a specific role in the etiology of DN.

Apo C-I is a protein constituent of chylomicrons, VLDL and HDL and while its precise physiological role is not well established, evidence has demonstrated support for its involvement in HDL metabolism through activation and inhibition of other proteins central to lipid metabolism, including CETP [51]. Association of rs4420638 with DN in T1D in this cohort has been previously reported [52].

Improved therapeutic regimens to lower LDL levels using statins have proved beneficial for patients both with and without diabetes with respect to CVD risk. In addition, increasing evidence suggests statins provide therapeutic benefit independent of cholesterol modulation, by improving endothelial and vascular function and reducing inflammation [53].

Common genetic loci explain only a proportion of the variation observed in lipid levels within the general population. Evidence in support of rare variants with potentially large individual effect size continues to grow, and is likely to make a significant impact on the genetic heritability of this condition [36]. Since our study focused only on common variants, untyped, highly penetrant rare variants in these genes could also contribute to DN. This study has insufficient power to detect rare variants particularly if the effect sizes are small in magnitude, such as the odds ratios of 1.2/1.3 which are more commonly found in common complex diseases (Table 4). Future amalgamation of independent cohorts with similar DN phenotypes will enable a more robust evaluation of such loci. In addition, other factors such as copy number variation or indeed epigenetic mechanisms (e.g. DNA methylation, histone modification and microRNAs) may also attenuate gene function affecting these pathways which modulate disease risk.

**Table 4:** Study power to detect various odds ratios for selected minor allele frequencies.

Odds	Minor Allele Frequency (MAF)			
ratio	0.10	0.20	0.30	0.40
1.2	30%	49%	59%	65%
1.3	56%	81%	89%	92%
1.4	79%	96%	99%	99%
1.5	93%	99%	100%	100%

Power calculations are based on 684 cases and 710 controls with odds ratio ranging from 1.2–1.5 for SNPs with a MAF between 0.10 and 0.40 with no correction for multiple testing.

Although the SNPs assessed in this study were chosen on the basis of previous associations with dyslipdemia there are a number of inherent limitations associated with using 53 SNPs across 37 genes [54]: (1) identification of association does not necessarily equate to functional significance given the concept of linkage disequilibrium (LD). (2) assessing one or two SNPs per gene may provide inadequate representation of the genetic architecture at that locus. (3) patterns of LD can vary significantly within and between different populations and therefore a significant association in one population may not necessarily translate across all populations.

In conclusion, we found no strong association between common variants in genes involved in dyslipidemia and DN. Further work to investigate lifestyle factors which influence genes may identify potential risk factors for susceptibility to DN.
